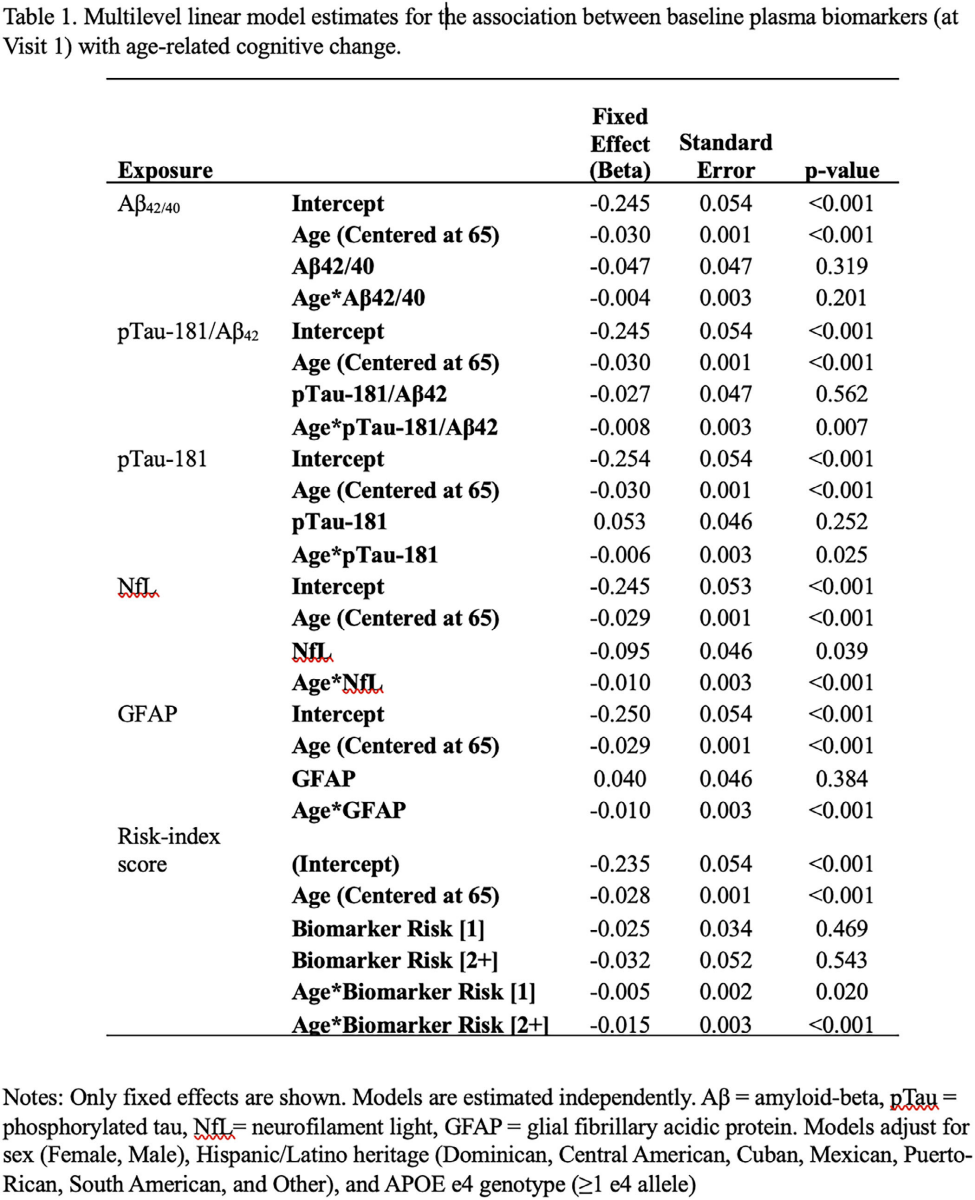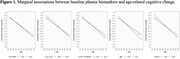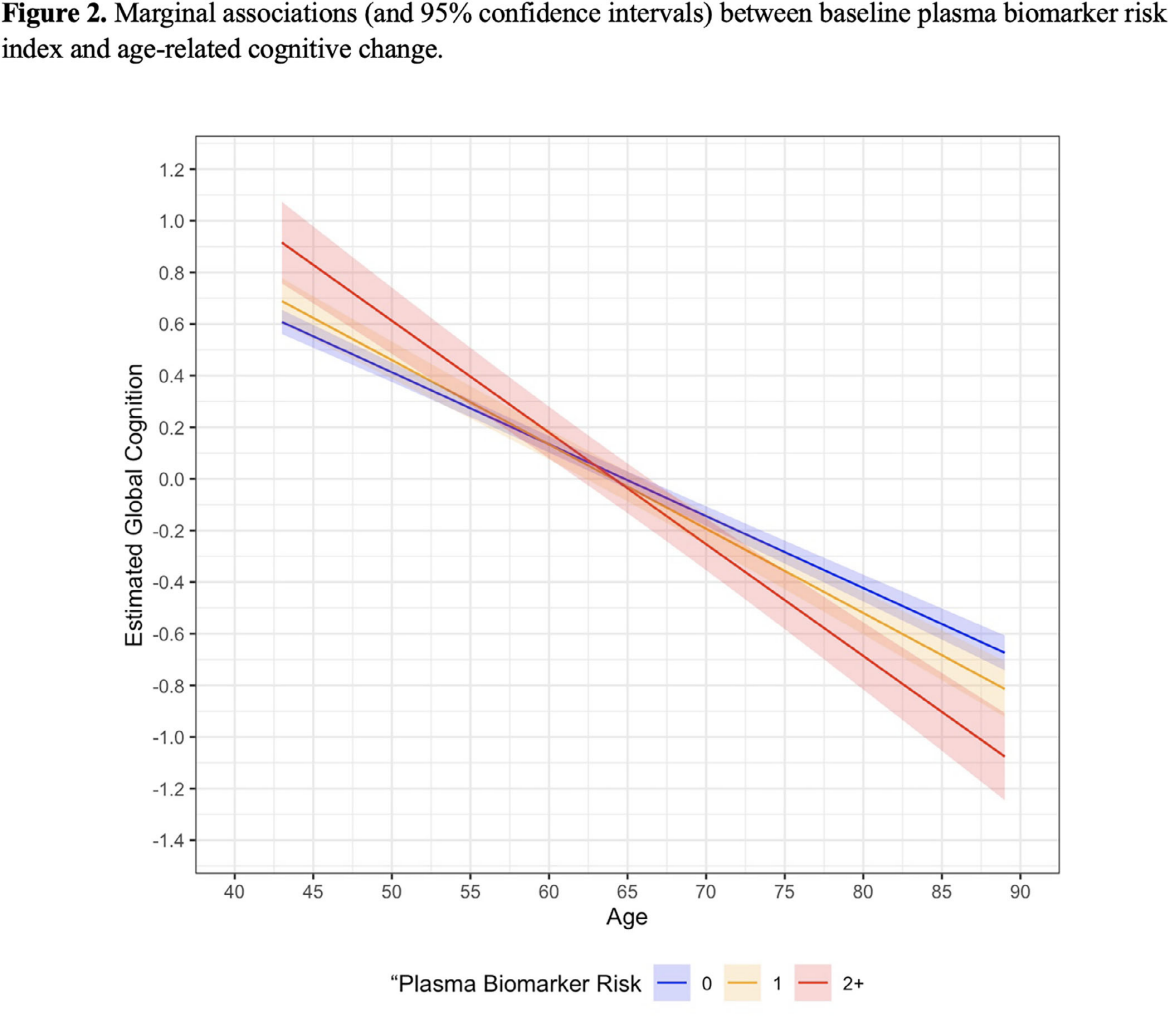# Association of plasma biomarkers for Alzheimer's disease with cognitive change among Hispanic/Latino adults: preliminary findings from HCHS/SOL and SOL‐INCA

**DOI:** 10.1002/alz70856_105946

**Published:** 2026-01-10

**Authors:** Freddie Márquez, Wassim Tarraf, Natasha Z. Anita, Deisha F Valencia, Fernando Daniel Testai, Humberto Parada, Carmen R Isasi, Paola Filigrana, Martha L Daviglus, Haibo Zhou, Linda C Gallo, Charles Decarli, Douglas R. Galasko, Bharat Thyagarajan, Hector M Gonzalez

**Affiliations:** ^1^ University of California, San Diego, La Jolla, CA, USA; ^2^ Wayne State University, Detroit, MI, USA; ^3^ University of Illinois at Chicago, College of Medicine, Chicago, IL, USA; ^4^ San Diego State University, San Diego, CA, USA; ^5^ Albert Einstein College of Medicine, Bronx, NY, USA; ^6^ University of Illinois at Chicago, Chicago, IL, USA; ^7^ University of North Carolina, Chapel Hill, Chapel Hill, NC, USA; ^8^ University of California, Davis, Davis, CA, USA; ^9^ Shiley‐Marcos Alzheimer's Disease Research Center, University of California, San Diego, CA, USA; ^10^ University of Minnesota, Minneapolis, MN, USA

## Abstract

**Background:**

The relationship between plasma biomarkers for Alzheimer's disease and longitudinal cognitive decline in diverse and underserved communities remains poorly understood. We investigated the associations between baseline plasma biomarkers and changes in cognitive performance over 13.5 years in a diverse cohort of Hispanic/Latino individuals.

**Method:**

We used data from the Hispanic Community Health Study/Study of Latinos (Visit 1; 2008‐2011) and the Study of Latinos‐Investigation of Neurocognitive Aging (SOL‐INCA; Visit 2; 2016‐2018) and SOL‐INCA2 (Visit 3; 2022‐2024) ancillary studies. We included 2,343 participants (aged 45‐74 years at Visit 1; 54.4 years on average) that completed their third visit of cognitive testing and had plasma samples analyzed (Quanterix Simoa HD‐X) for amyloid‐beta (Aβ_42/40_), phosphorylated tau‐181 (pTau‐181), neurofilament light chain (NfL), and glial fibrillary acidic protein (GFAP) at Visit 1. Global cognitive performance was a standardized average composite of four z‐scored cognitive tests relative to the baseline mean and standard deviation. The plasma biomarker measures were dichotomized to focus on high‐risk groups (bottom 10^th^ percentile for Aβ_42/40_; top 10^th^ percentile for pTau‐181, pTau‐181/Aβ_42_, NfL, and GFAP). Additionally, we used a risk‐index score based on the count of plasma biomarker (excluding pTau‐181/Aβ_42_) measures that are high‐risk (0,1, and ≥2). We used multilevel linear models to examine cognitive aging trajectories with models covarying for sex, Hispanic/Latino background, and *APOE* e4 genotype.

**Result:**

In each model, age was associated with decrements in global cognitive change (Table 1). Individuals in the top 10^th^ percentile for pTau‐181, pTau‐181/Aβ_42_, NfL, and GFAP had more pronounced age‐related declines in global cognitive performance (Figures 1 and 2). We observed no differences in age‐related global cognitive change for individuals in the bottom 10^th^ percentile for Aβ_42/40_. Individuals with higher risk (≥2 biomarkers in the high‐risk groups) had more pronounced age‐related cognitive decline.

**Conclusion:**

Preliminary findings suggest plasma biomarkers for pTau‐181, pTau‐181/Aβ_42_, NfL, and GFAP predict cognitive decline before symptoms manifest. Individuals with high levels of these biomarkers are at elevated risk for age‐related cognitive decline. The threshold for differences in the trajectories varies by biomarker and differences in trajectories are more apparent in older age suggesting the likelihood for nonlinear change.